# Dysregulated Immunity in Pulmonary Hypertension: From Companion to Composer

**DOI:** 10.3389/fphys.2022.819145

**Published:** 2022-02-17

**Authors:** Teresa C. Funk-Hilsdorf, Felix Behrens, Jana Grune, Szandor Simmons

**Affiliations:** ^1^Junior Research Group “Immunodynamics”, Institute of Physiology, Charité – Universitätsmedizin Berlin, Berlin, Germany; ^2^Laboratory of Lung Vascular Research, Institute of Physiology, Charité – Universitätsmedizin Berlin, Berlin, Germany; ^3^Berlin Institute of Health (BIH), Berlin, Germany; ^4^DZHK (German Centre for Cardiovascular Research), partner site Berlin, Berlin, Germany

**Keywords:** pulmonary hypertension, autoimmunity, inflammation, innate and adaptive immune cells, S1P, complement, cytokines

## Abstract

Pulmonary hypertension (PH) represents a grave condition associated with high morbidity and mortality, emphasizing a desperate need for innovative and targeted therapeutic strategies. Cumulative evidence suggests that inflammation and dysregulated immunity interdependently affect maladaptive organ perfusion and congestion as hemodynamic hallmarks of the pathophysiology of PH. The role of altered cellular and humoral immunity in PH gains increasing attention, especially in pulmonary arterial hypertension (PAH), revealing novel mechanistic insights into the underlying immunopathology. Whether these immunophysiological aspects display a universal character and also hold true for other types of PH (e.g., PH associated with left heart disease, PH-LHD), or whether there are unique immunological signatures depending on the underlying cause of disease are points of consideration and discussion. Inflammatory mediators and cellular immune circuits connect the local inflammatory landscape in the lung and heart through inter-organ communication, involving, e.g., the complement system, sphingosine-1-phosphate (S1P), cytokines and subsets of, e.g., monocytes, macrophages, natural killer (NK) cells, dendritic cells (DCs), and T- and B-lymphocytes with distinct and organ-specific pro- and anti-inflammatory functions in homeostasis and disease. Perivascular macrophage expansion and monocyte recruitment have been proposed as key pathogenic drivers of vascular remodeling, the principal pathological mechanism in PAH, pinpointing toward future directions of anti-inflammatory therapeutic strategies. Moreover, different B- and T-effector cells as well as DCs may play an important role in the pathophysiology of PH as an imbalance of T-helper-17-cells (T_H_17) activated by monocyte-derived DCs, a potentially protective role of regulatory T-cells (T_reg_) and autoantibody-producing plasma cells occur in diverse PH animal models and human PH. This article highlights novel aspects of the innate and adaptive immunity and their interaction as disease mediators of PH and its specific subtypes, noticeable inflammatory mediators and summarizes therapeutic targets and strategies arising thereby.

## Introduction

Pulmonary hypertension (PH) is a severe pathologic condition characterized by elevated mean pulmonary arterial pressure (mPAP) of ≥20 mmHg at rest measured by right heart catheterization (Simonneau et al., 2019). Clinically, PH in earlier stages causes only few symptoms that appear rather unspecific delaying the actual time point of diagnosis. Observable symptoms may be dyspnoea, fatigue, syncope on exertion, and edema (Hoeper et al., 2017). However, progression of the disease frequently leads to right ventricular (RV) dysfunction causing volume overload, and, thus, is accompanied by high mortality (Hoeper et al., 2016). Pathophysiological hallmarks of PH are lung vascular remodeling evident in small to medium-sized distal pulmonary arteries and endothelial dysfunction (Kurakula et al., 2021). Particularly, the subsequently enhanced pulmonary vascular resistance results in increased RV afterload, leading to RV hypertrophy and, ultimately, death by RV failure (Bogaard et al., 2009). As of yet, five PH classes have been defined based on their clinical presentation, pathophysiology, hemodynamic characteristics, and treatment responses (Kovacs et al., 2018). Group 1, for example, comprises patients who suffer from pulmonary arterial hypertension (PAH), which, in turn, is based on various etiologies: idiopathic, heritable, drug- and toxin-induced or secondary due to particular conditions like connective tissue disease, HIV infection, portal hypertension, congenital heart disease, schistosomiasis, and long-term response to calcium channel blockers (Simonneau et al., 2019). Considering the underlying disease, the different PH types show a remarkable variance in their prevalence: while only 15 cases/1 million adults are described for idiopathic pulmonary arterial hypertension (iPAH, group 1 PH), patients with left heart disease (LHD) develop the most common type of PH (PH-LHD, group 2 PH) and comprise the largest set of PH patients although the estimated prevalence of PH in patients with heart failure relies on diagnostic criteria and ranges from 25% to 83% (Charalampopoulos et al., 2018). LHD and subsequent PH may be initially caused by valvular heart disease, loss of viable myocardium (e.g., myocardial infarction, MI), or chronic heart failure (HF; Galie et al., 2016). PH was reported as a frequent complication in both types of HF, HF with reduced ejection fraction (HFrEF) and HF with preserved EF (HFpEF), but was observed more frequently (40% vs. 83%) and more severe in HFpEF patients (Lam et al., 2009). Recent data suggest that not only the incidence but also the pulmonary vascular pathophysiology and the resulting RV outcomes are distinct between HFpEF and HFrEF with more pronounced lung vascular remodeling in HFpEF (Fayyaz et al., 2018). The reasons why certain types of LHD are more prone to develop PH remain elusive and, unlike PAH, no efficient therapies for either form of PH-LHD have been approved. As a consequence, the mean time to death from first echocardiographic diagnosis of PH-LHD has been reported with only 4.1 years demonstrating the need for deeper mechanistic insights and the development of novel treatment options for PH-LHD (Strange et al., 2012).

Pulmonary vascular remodeling is a hallmark of all forms of PH, involving a multitude of structural changes in the pulmonary vasculature. Characteristics of pulmonary blood vessel remodeling are thickening and strengthening of the intimal and/or medial muscular vessel layers. In addition, cells expressing smooth muscle-specific markers in pre-capillary arterioles, resulting from proliferating pulmonary arterial smooth muscle cells (PASMCs) and cellular trans-differentiation (endothelial-mesenchymal transformation). In severe forms of PAH, disease progression leads to vaso-occlusive lesions, involving endothelial cells (ECs), PASMCs, and stromal cells of non-vascular origin (Shimoda and Laurie, 2013). All together these processes result in shrinkage of the vascular lumen diameter, reduction of vasodilatory capacity, and increased pulmonary vascular resistance, ultimately resulting in sustained PH. Depending on the type of PH, the development of vessel remodeling takes place either in an idiopathic fashion or secondary to other underlying conditions, e.g., in PH-LHD, in which chronically elevated intravascular pressure causes pulmonary vascular remodeling (Breitling et al., 2015). Of note, pulmonary vascular remodeling is not solely attributable to passive congestion but involves active remodeling of the pulmonary vasculature. Cumulative evidence suggests that inflammation in general modulates disease progression and active recruitment of innate immune cells, constitute key pathogenic drivers of vascular remodeling in PH (Stacher et al., 2012; Zelt et al., 2019).

In fact, various compartments and cells of the innate and adaptive immune system have been studied regarding their involvement in PH pathophysiology, especially PAH. Increased levels of inflammatory cells have been detected in the periphery of remodeled pulmonary vasculature while the amount of accumulating immune cells positively correlated with the intensity of remodeling (Stacher et al., 2012; Marsh et al., 2018). Inflammatory cells are able to release lipid mediators, chemokines, and cytokines, promoting the recruitment of further immune cells, which then leads to manifestation of the inflammatory state. Therefore, chemokines and cytokines are also of particular interest referring to their contribution to inflammation and vascular remodeling in PH (Marsh et al., 2018). Dysregulated immunity appears to be an important mechanism driving disease development and thus holds great potential for novel therapeutic approaches, especially considering the recent success of targeted anti-inflammatory therapies in autoimmune diseases like anti-tumor necrosis factor-*α* (TNF-α) therapy with infliximab in rheumatoid arthritis (Lipsky et al., 2000) or even approaches of anti-inflammatory therapy in atherosclerotic disease targeting interlukin-1β (Ridker et al., 2017). As of yet, therapeutic options of PH are rather limited to vasodilatation to diminish RV afterload (Rabinovitch et al., 2014; Marsh et al., 2018; Goldenberg et al., 2019; Hu et al., 2020).

An advanced perception of the causal immunological relations in PH could not only lead to the development of effective targeted therapies but also to the early identification of biomarkers facilitating the diagnosis of PH or indicating disease severity, eventually resulting in improved disease outcome. Reflecting on these circumstances, this review discusses the interplay and contribution of innate and adaptive immunity to the pathophysiology of PH, especially PAH and PH-LHD, and sums up potential therapeutic targets considering immunological deviations in the lung and heart.

## Disease-Associated Humoral Mediators of Inflammation

### Chemokines and Interleukins as Indicators of PH Prognosis

Chemokines and interleukins play vital roles in both initiation and maintenance of inflammation (Oppenheim et al., 1991). Chemokines comprise a large family of small-sized cytokines that are able to trigger leukocyte migration and recruitment, promote angiogenesis and vascular remodeling by targeting their respective G-protein coupled receptor (Oppenheim et al., 1991). Indeed, due to their chemotactic ability and thus interconnection of different immune cells, which in turn vary in their expression of chemokine receptors, chemokines seem to be key players in inflammatory processes (Oppenheim et al., 1991). Although most chemokines are considered as pro-inflammatory, some rather endorse homeostasis and are involved, e.g., in morphogenesis (Raz and Mahabaleshwar, 2009). Interleukins belong to the family of cytokines are glycoproteins and primarily accountable for conveying communication between lymphocytes, monocytes, and macrophages (Strober and James, 1988).

**Figure 1:**
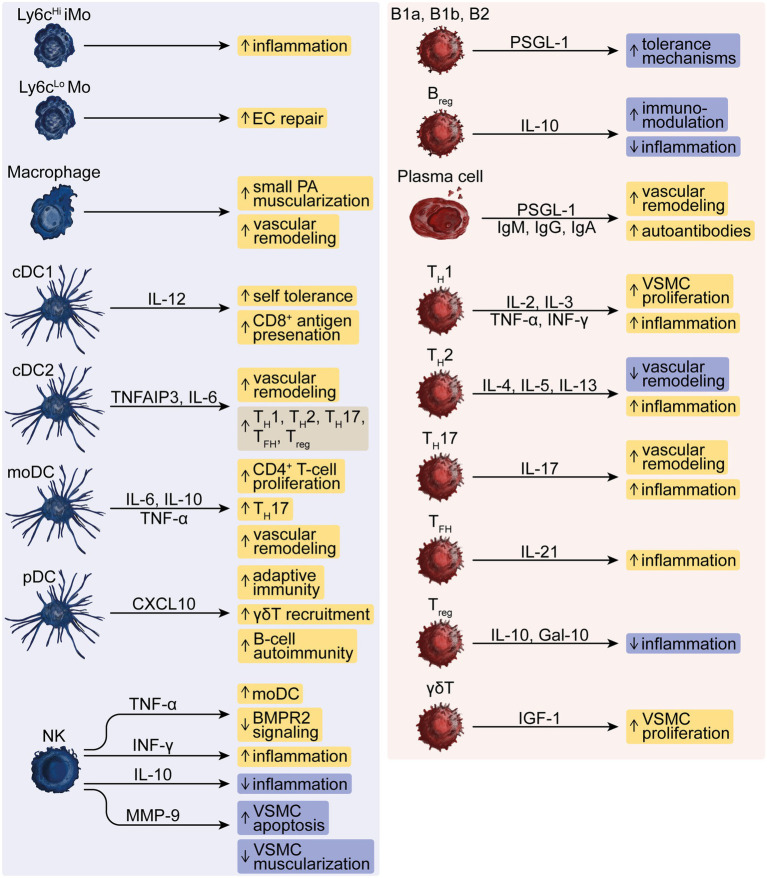
The dysregulated function of innate and adaptive immune cells in PH. The innate effector cells represented by Ly6c^Hi^ inflammatory monocytes (iMo) and Ly6c^Lo^ monocytes (Mo), macrophages, conventional type-1 or type-2 dendritic cells (cDC1 or cDC2), monocyte-derived DCs (moDC), plasmacytoid DCs (pDC) and natural killer (NK) cells drive, e.g., inflammation, vascular remodeling and autoimmunity and modulate responses by B-lymphoid cells such as innate-like B1a and B1b, conventional B2 and regulatory B-cells (B_reg_), T-helper (T_H_)-1, −2, −17 cells, T follicular helper (T_FH_) cells, regulatory T (T_reg_) -cells and *γ*δT-cells that can further aggravate these responses in PH. Pro- and anti-inflammatory mediators comprise interleukins (IL), TNF-*α*, IFN-γ, CXC chemokines, matrix metallopeptidase (MMP)-9, Galectin (Gal)-10, and insulin-like growth factor (IGF)-1 as well as autoreactive immunoglobulins (Ig)-M, −G, and -A, and P-selectin glycoprotein ligand (PSGL)-1.

Several studies showed that the development and progression of PH are associated with the dysregulated expression of several chemokines and chemokine receptors in the pulmonary vasculature and, consequently, with the recruitment of immune cells, proliferation of PASMCs and endothelial dysfunction (Koudstaal et al., 2021; Figure 1). Clinically, chemokines and interleukins attracted considerable attention in human PH serving as inflammatory biomarker. In fact, in PAH patients, elevated serum levels of IL-6, IL-8, and IL-10 were associated with reduced survival (Soon et al., 2010; Matura et al., 2015; Table 1). Evidence arises that IL-6 incorporates key parts in the dysregulated immunity in PH since upregulation of IL-6 and IL-6 receptor (IL-6R) causes media thickening of the pulmonary vasculature in iPAH (Tamura et al., 2018) and IL-6-overexpressing mice spontaneously develop PH under chronic hypoxia (Steiner et al., 2009).

**Table 1:** Summary of the most important humoral mediators of perivascular inflammation in pulmonary hypertension (PH) and findings regarding natural killer (NK) cells.

Effector	Patient cohort/animal model	Effect	Mortality	References
Chemokines/cytokines	PAH	Elevated serum IL-6, IL-8, and IL-10	**↑**	Soon et al., 2010; Matura et al., 2015
	PAH	Elevated serum CCL5, CCL7, CCL4, MIF, TNF-*β*, CXCL9, IL-18, IL-3, and TRAIL	**↑**	Sweatt et al., 2019
Complement	Hx PH mice	Increased expression of C5a and C3a receptor perivascularly, Cfb elevated	N/A	Frid et al., 2020
NK cells	Inflammatory heart disease	Decreased CD56^bright^ and CD56^dim^ circulating NK cells, reduced IFN-γ	N/A	Hak et al., 2007
	PAH	Decreased CD56^dim^/CD16^+^ NK cells, decreased expression of NKp46 and KIR3DL1	N/A	Ormiston et al., 2012
	CTD-PAH	NK cell and CD8^+^ T cell depletion	**↑**	Edwards et al., 2013
	NK cell-deficient Nfil3^−/−^ mice	IL-23 elevation, RV hypertrophy, RVSP increased, and muscularization pulmonary arteries	N/A	Ratsep et al., 2018
	SU-Hx PAH rats	RV dysfunction, downregulated NK-mediated genes in RV	N/A	Suen et al., 2019

*CCL4, C-C-chemokine-ligand-4; CCL5, C-C-chemokine-ligand-5; CCL7, C-C-chemokine-ligand-7; Cfb, complement factor b; CTD-PAH, connective tissue diease associated pulmonary hypertension; CXCL9, C-X-C motif chemokine ligand 9; Hx, hypoxia; IFN-γ, Interferon γ; IL, interleukin; KIR3DL1, inhibitory killer immunoglobulin-like receptor 3DL1; MIF, macrophage migration inhibitory factor; N/A, not applicable; NK, natural killer; PAH, pulmonary arterial hypertension; PH, pulmonary hypertension; RV, right ventricle; RVSP, right ventricular systolic pressure; SU-Hx, Sugen5416 hypoxia model; TNF-β, Tumor necrosis factor β; TRAIL, Tumor necrosis factor-related apoptosis-inducing ligand*.

In line with these results, Koudstaal et al. demonstrated increased plasma levels of IL-6 and C-X-C motif chemokine ligand (CXCL)-9 and CXCL13 in connective tissue disease-associated (CTD)-PAH patients compared to healthy controls (Koudstaal et al., 2021). While CXCL9 is engaged in T_H_1 differentiation (Groom et al., 2012), CXCL13 is important for B-cell arrangement in germinal centers (Olsson et al., 2016), stressing a vital role of T- and B-lymphocytes in the pathogenesis of PH. Moreover, in patients with iPAH levels of tumor growth factor-β (TGF-β), IL-10, and CXCL9 were increased (Koudstaal et al., 2021), whereas in patients with systemic sclerosis (SSc)-associated PAH plasma levels of TNF-*α*, that is mainly released by macrophages, was elevated compared to those patients who suffered from SSc but did not develop PAH (Kylhammar et al., 2018), revealing varying characteristic of increased chemokine release that might depend on the underlying PH subtype. Sweatt et al. identified a proteomic panel of 48 cytokines, chemokines, and factors of PAH patients resulting in different proteomic profiles. Interestingly, when taking distinct patterns of elevated cytokines into account, the accompanied elevation of C-C-chemokine-ligand-5 (CCL5), C-C-chemokine-ligand-7, C-C-chemokine-ligand-4 (CCL4), macrophage migration inhibitory factor (MIF), TNF-β, CXCL9, IL-18, IL-3, and tumor necrosis factor-related apoptosis-inducing ligand (TRAIL) was associated with high mortality within 5 years after recruitment (Sweatt et al., 2019; Table 1). Of note, TRAIL affects migration and proliferation of SMCs in iPAH, while anti-TRAIL antibodies prevent vascular remodeling in PAH models (Hameed et al., 2012). MIF, CCL5, and IL-18 are involved in recruitment of mononuclear cells to the endothelium and, therefore, support a pro-inflammatory condition (Ross et al., 2012; Le Hiress et al., 2015).

However, limited information is present on the clinical and prognostic impact of altered chemokine or cytokine levels in patient serum, especially considering the different PH types and their subgroups in the studies conducted so far. Nonetheless, correlating chemokine and cytokine levels in a range of PH patients, referring to them as inflammatory makers, with clinical parameters might allow to define a concrete disease prognosis and perhaps to even cluster in between the diverse groups of PH in the future.

### Sphingosine-1-Phosphate

Sphingolipids exert a fundamental role in the cardiovascular system by orchestrating cell migration, angiogenesis, homeostasis, and physiology. Therefore, a comprehensive understanding of sphingolipid pathobiology gains persistent interest in translational medicine. Sphingosine-1-phosphate (S1P), one of the best-characterized bioactive lipids and potent signaling molecule, mediates its biological function on its target T-cell in the hematopoietic and vascular systems by five different G-protein-coupled receptors, named S1P receptors (S1PR1-5). An extensive body of work has described the multifunctional impact of the S1P/S1PR signaling axis on immune cell migration, angiogenesis, and barrier integrity in the context of cell development, and physiological and pathological conditions (Schwab and Cyster, 2007; Cartier and Hla, 2019).

#### S1P Synthesis and Release

**Figure 2:**
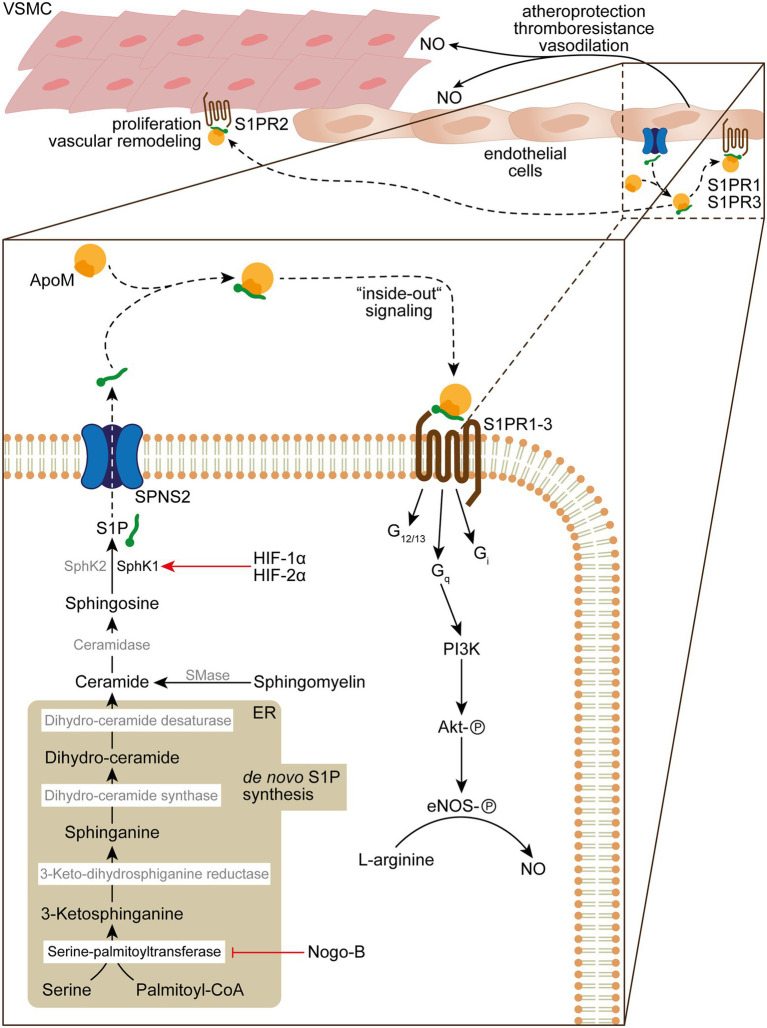
*De novo* sphingolipid biosynthesis, S1P “inside-out” signaling and its role in vascular homeostasis and function. *De novo* sphingolipid biosynthesis is initiated in the endoplasmatic reticulum (ER) by condensation of serine and palmitoyl-CoA into 3-ketosphinganine by serine-palmitoyltransferase, which activity can be inhibited by Nogo-B. 3-ketosphinganine is rapidly metabolized into sphinganine by 3-keto-dihydrosphinganine reductase. Subsequently, sphinganine is converted by dihydro-ceramide synthase into dihydro-ceramide, which is desaturated by dihydro-ceramide desaturase into ceramide. The ceramide pool can further be increased by sphingomyelinase (SMase) that catabolizes sphingomyelin in the plasma membrane, which is transformed by ceramidase into sphingosine. In the final step of the S1P biosynthesis sphingosine is phosphorylated by SphK1/SphK2 in order to form S1P, which is released by secretion through SPNS2. Upon release S1P binds to the HDL ApoM (~65%) or albumin/Apo4 (~35%), which delivers S1P to S1PRs. Autokrine S1P/S1PR1 and − 3/G_q_ “inside-out” signaling results in activation of Akt by phosphorylation through PI3K and subsequent activation of eNOS that converts L-arginine to NO, which fosters atheroprotection, thromboresistance and vasodilatation. In addition, S1PR2 signaling can result in VSMC proliferation and vascular remodeling in PH.

Extracellular S1P gradients and the consecutive S1PR signaling were identified as critically important in cardiovascular physiology and pathophysiology. Indeed, tissue- and cell-specific secretion, chaperone association, and extracellular metabolism achieve the establishment of an S1P gradient. S1P is the metabolic product of the *de novo* sphingolipid synthesis pathway, by which ceramide is formed through the sequential action of serine palmitoyl-transferase, 3-keto-dihydrosphinganine reductase, and dihydro-ceramide synthases from cytosolic serine and palmitoyl-CoA molecules (Figure 2). Further deacetylation of ceramide in the Golgi apparatus by ceramidases forms sphingosine that subsequently is converted by sphingosine-kinase (SphK)1 or −2 to S1P (Maceyka and Spiegel, 2014; Figure 2). Robust intracellular generation of S1P by SphK1 and SphK2 and release by the S1P-transporter major facilitator superfamily domain-containing protein 2B (MFSD2B) from erythrocytes and platelets (Vu et al., 2017; Kobayashi et al., 2018), and the S1P-transporter spinster-homologue-2 (SPNS2) from blood and lymphatic ECs (Fukuhara et al., 2012; Simmons et al., 2019) are necessary to counteract degradation of S1P in the interstitial spaces by the S1P lyase (Kharel et al., 2011) or the lipid phosphate phosphohydrolase 3 (LPP3; Ramos-Perez et al., 2015; Figure 2). The high turnover of S1P in the interstitial space contributes to the formation of a S1P gradient between the lumen and the extravascular space that, in turn, is sensed by S1PR on hematopoietic cells and facilitates immune cell trafficking (Kharel et al., 2011). S1P is associated to chaperone proteins such as apolipoprotein M (ApoM, ~65%; Christoffersen et al., 2011) or to a much lower extent also to serum albumin and ApoA4 (Obinata et al., 2019) in the blood and lymph (Fleming and Wojciak, 2018) that warrant S1PR activation on vascular recipient T-cells such as endothelial and vascular smooth muscle cells (VSMCs), which contributes to vascular health by, e.g., endothelial nitric oxide synthase (eNOS) activation (Christoffersen et al., 2011; Figure 2).

#### Sphingosine-Kinase-1 and -2

Given the complexity of S1P metabolism, secretion, spatial distribution, and S1PR signaling on vascular integrity, various molecular targets in sphingolipid biology gain attention in PH. One hallmark of PH is extensive vascular remodeling, characterized by vascular endothelial dysfunction and disordered VSMC proliferation and migration, leading to increased pulmonary vascular resistance that increases the risk for fatal right heart failure. It was shown that S1P associates with elevated levels of SphK1 in PASMCs in those experimental models (Chen et al., 2014). Of note, it was demonstrated that SphK1 is regulated by hypoxia-inducible factor-1α (HIF-1α) and -2α (HIF-2α), which play a fundamental role in the pathogenesis of various forms of PH (Pullamsetti et al., 2020) and, in fact, has respective HIF-responsive elements in the promoter region of SphK1 (Schwalm et al., 2008; Figure 2). Thus, genetic deletion of SphK1 and pharmacological inhibition of SphK1 protected against hypoxia-induced hypertension (Chen et al., 2014). By contrast, SphK2 seems to play a minor role in the context of PH, as SphK2 protein levels were unaffected in PAH patients and under experimental hypoxic conditions (Chen et al., 2014). Given the elevated levels of SphK1 in PASMCs and lungs of PAH patients, it is likely that S1P stimulates PASMC proliferation. Indeed, abrogation of S1PR2 signaling by siRNA knockdown or by use of the S1PR2/S1PR4 antagonist JTE013 prevented and reversed PASMC proliferation and reduces right ventricular systolic pressure (RVSP) under hypoxic conditions *in vivo* (Chen et al., 2014). This appears reminiscent of an “inside-out” signaling mechanism where SphK1 activity leads to the production and release of S1P that stimulates PASMC proliferation through autocrine S1PR signaling (Takabe et al., 2008; Figure 2).

#### Nogo-B Regulation of Sphingolipid Synthesis

Besides VSMCs, ECs are also a target of S1P in the vascular microenvironment and an important and tightly regulated source of plasma S1P (Fukuhara et al., 2012). Cantalupo et al. could elegantly show that the Rtn4-protein-family member Nogo-B regulates the *de novo* sphingolipid biosynthesis in ECs and VSMCs (Cantalupo et al., 2015). Nogo-B is located in the endoplasmic reticulum where it inhibits the serine palmitoyltransferase and, therefore, blocks the initial condensation of serine and palmitoyl-coenzyme A to 3-ketosphinganine toward the *de novo* biosynthesis of ceramides and S1P (Cantalupo et al., 2015; Figure 2). It was demonstrated that treatment with the S1PR1 agonist SEW2871 restores normal blood pressure in hypertensive mice (Cantalupo et al., 2015). Indeed, the autocrine S1P/S1PR1/eNOS signaling pathway in Nogo-B deficient mice prevents the onset of experimental hypertension (Cantalupo et al., 2015). Endothelium-derived nitric oxide (NO) critically mediates vascular relaxation and has been implicated in blood pressure regulation (Igarashi and Michel, 2008; Figure 2). Thus, the activation of eNOS by autocrine S1P/S1PR1 signaling is of critical importance for preserving vascular function (Figure 2). In line with this notion, the chronic treatment of normotensive mice with fingolimod (FTY720), a functional antagonist of S1PR1, S1PR3, S1PR4, and S1PR5, led to increased blood pressure and aggravated hypertensive responses in Angiotensin-II-treated mice in comparison to the vehicle-treated mice (Cantalupo et al., 2017). Further, the observation of increasing blood pressures in multiple sclerosis patients treated orally with fingolimod (Comi et al., 2010) highlight the anti-hypertensive functions of S1PR1 signaling.

#### SPNS2

The relevance of endothelial derived S1P for hypertension has recently been addressed in greater detail by analyses using endothelial-cell specific Spns2-KO (ECKO-Spns2) mice to investigate the consequence of impaired S1P-secretion from ECs on endothelial dysfunction (Del Gaudio et al., 2021; Figure 2). Due to the impaired release of S1P from ECs into the blood circulation, ECKO-Spns2 mice showed significantly reduced plasma S1P levels, which correlated with elevated blood pressure at baseline and aggravated cardiac hypertrophy (Del Gaudio et al., 2021). Importantly, binding of endothelial-derived S1P to ApoM in the plasma seems to be necessary for S1P’s effect as blood pressure regulator, since ApoM-deficient mice also exhibited a hypertensive phenotype (Del Gaudio et al., 2021) and administration of a recombinant soluble ApoM-Fc fusion protein reduced blood pressure in hypertensive mice (Swendeman et al., 2017; Figure 2). Of note, administration of ApoM-Fc, which functions as a carrier of S1P, promoted endothelial S1PR1-signaling with minor effects on lymphocyte migration, which underlines its therapeutic potential in cardiovascular disease (Swendeman et al., 2017).

#### Leukotriene B4

Interestingly, there seems to be an interplay of innate immunity and S1P biology since myeloid cells can induce the activation of SphK1 and promote eNOS activity. It was demonstrated that macrophages are the predominant source of leukotriene B4 (LTB4), which was found elevated in blood of PAH patients and in the bronchoalveolar lavage fluid of athymic SU5416-treated PH rats (Tian et al., 2013). Macrophage-derived LTB4 diminishes “inside-out” S1P-signaling on PAECs by reducing SphK1 levels and concomitant S1P production and autocrine S1PR1 activation. The resulting failure to activate eNOS culminates in endothelial dysfunction (Tian et al., 2013). However, the functional role of LTB4 in PH is complex. Although LTB4 aggravates PAH through the activation of pulmonary artery adventitial fibroblasts (Qian et al., 2015), phase 2 of the LIBERTY study (NCT0266455) failed to proof an impact of the reversible protease inhibitor Ibenimex (Bestatin), a blocker of the leukotriene A4 hydrolase that converts LTA4 to LTB4, on pulmonary vascular resistance or exercise capacity (Melão, 2018). Nevertheless, bone morphogenic protein receptor-2 (BMPR2) haploinsufficient rats transduced with 5-lipoxygenase (5-LO), which catalyzes the formation of LTA4 that is hydrolyzed to LTB4, develop severe PAH in frequencies comparable to human BMPR2 mutations (Tian et al., 2019). Furthermore, BMPR2 mutations in humans and rats with PAH result in expression of non-viral 5-LO expression in the neointima (Tian et al., 2019), suggesting that inhibition of the disturbed “inside-out” signaling pathway through LTB4 may be implemented by S1PR1 agonist therapy in PAH patients with BMPR2 mutation. The increased systolic blood pressure in endothelial-cell specific S1PR1-KO mice and blood pressure-reducing effects of the S1PR1 agonist SEW2871 in hypertensive mice further support this promising therapeutic avenue (Cantalupo et al., 2015, 2017).

### Dysregulated Complement Signaling

The complement system is a crucial part of the innate immune system and can be divided into three activation pathways, namely the classical pathway, the mannose-binding lectin pathway and the alternative pathway. While all three of these pathways get activated through diverse initiators like immune complexes, apoptotic cells or pathogens itself, the complement system as a whole plays a fundamental role in defense against infections, interconnecting innate and adaptive immunity and eliminating immune complexes (Walport, 2001). Depending on the activation pathway, one can divide into the proteins C1 through C9 representing the classical pathway and the proteins of the alternative pathway, which are called “factors.” The soluble complement proteins are mainly produced in hepatocytes and macrophages but also fibroblasts and ECs can contribute to their formation (Lubbers et al., 2017). In the course of the activation, the majority of the complement proteins are zymogens, and as such activate themselves by proteolytic cleavage, e.g., C4 cleaves into C4a and C4b. The terminal point of the pathways is the formation of so-called membrane-attack complexes. Of note, there exist various regulatory mechanisms. Exemplarily, C3 is constantly hydrolyzed and thus activates the alternative pathway. To regulate this activation, C3b is inhibited by factor H (FH) that is functioning in the cleavage of C3b and thus prevents ongoing and spontaneous alternative pathway activation (Haapasalo and Meri, 2019). The importance of these control mechanisms is displayed by the multiple disorders that can emerge upon dysregulation of the complement system. Complement hyperactivation can lead to autoinflammatory conditions: an inaccurate regulation of C3 and a factor H deficiency are for example associated with glomerulonephritis and C1 inhibitor deficiency can lead to hereditary angioedema (Walport, 2001). Apart from these pathologies, evidence arises that the complement system also participates in the pathophysiology of diverse pulmonary ailments like acute lung injury and PAH (Cleary et al., 2020; Frid et al., 2020).

#### Activation of the Alternative Pathway

Recent work in PAH animal models and on human PH lung tissue showed an activation of the complement system—in detail, an increase of perivascular cells expressing C5a and C3a receptors (Frid et al., 2020). Further, mRNA analyses of these mice lungs revealed elevated levels of the alternative pathway activator Complement factor B (Cfb; Frid et al., 2020). It also became evident, that Cfb and C5 deficient mice did not present perivascular recruitment of macrophages after being exposed to hypoxia, showing a substantial contribution of the alternative pathway to perivascular inflammatory processes in general and particularly leukocyte infiltration in PAH (Frid et al., 2020; Table 1).

#### GM-CSF and Complement Activation

The recruitment of monocytes and macrophages relies on chemokines and cytokines, for example, granulocyte-macrophage colony-stimulating factor (GM-CSF), which has been studied in the context of reduced expression of BMPR2 in iPAH. Actually, GM-CSF is produced by various cells, e.g., epithelial cells in the lung or T-helper (T_H_) cells, and was initially outlined as growth factors during myelopoiesis. Today GM-CSF is known beyond that as pleiotropic factor promoting the initial differentiation of myeloid precursors into neutrophils, monocytes, macrophages, and dendritic cells (DCs), controlling cell proliferation, and activation of phagocytes and is widely discussed to play a role in inflammation and autoimmune conditions like multiple sclerosis (MS) or rheumatoid arthritis by inducing inflammation *via* monocytes (Williamson et al., 1988; Carrieri et al., 1998; Burmester et al., 2011; Croxford et al., 2015; Becher et al., 2016).

Interestingly, the production of GM-CSF can result from complement activation, indicating in turn an activation of innate immune compartments through the complement system (Sawada et al., 2014; Frid et al., 2020). Sawada et al. could show that reduced BMPR2 expression is associated with elevated GM-CSF levels in the lungs of iPAH patients, which, in turn, trigger monocyte and macrophage recruitment and, thus, inflammation leading to disease progression (Sawada et al., 2014). Loss of function mutations in the gene encoding BMPR2 is the main heritable risk factor for developing PAH (Austin and Loyd, 2014). The debatable impact of a potential interplay between decreased BMPR2 expression, complement activation and subsequent augmentation of GM-CSF and, thus, perivascular inflammation in PH have yet to be elucidated.

#### B-Cell Immunity

In a recent study, Frid et al. further depict an interconnection between the alternative complement pathway activation and perivascular accumulation of IgM and IgG antibodies (Frid et al., 2020); therefore, raising awareness of the interaction and a potential causal chain of dysregulated B-cell homeostasis and complement pathways leading to PH. In fact, complement activation through immunoglobulins seems to have a regulatory function in terms of proliferation and inflammation in models of PH due to hypoxia. In fact, mice lacking all circulating immunoglobulins seemed protected from hypoxia-induced perivascular cell proliferation in the lung defined by reduced numbers of Ki67^+^ cells and CDk1 expression (Frid et al., 2020). They further featured fewer perivascular accumulations of complement C3 and CD68^+^ macrophages when compared to hypoxia wildtype (WT) mice. Reconstitution with IgG restored the hypoxia WT phenotype, stressing the fundamental involvement of IgG to perivascular inflammation in PH. Interestingly, particular plasma complement patterns can act as a determinant of disease outcome in PAH patients (Frid et al., 2020). This is why inhibition of complement activation targeting specific components could serve as a therapeutic strategy, which is indeed element of recent studies that engage with complement inhibitor therapy in autoimmune diseases like myasthenia gravis (Albazli et al., 2020).

### Nicotinamide Phosphoribosyltransferase

Nicotinamide phosphoribosyltransferase (NAMPT) is also known as pre-B-cell colony enhancing factor 1 (PBEF1) or visfatin and possesses various functions depending on its extracellular or intracellular deposit (Revollo et al., 2007; Gao et al., 2019). Intracellularly, it works as the key enzyme catalyzing the biosynthesis of the redox co-enzyme nicotinamide adenine dinucleotide (NAD; Sommer et al., 2008; Audrito et al., 2020).

NAMPT plays a role in the regulation of cellular metabolism and cell proliferation and modulates the activation of NAD-induced enzymes (Sommer et al., 2008; Grahnert et al., 2011). On the other hand, extracellular NAMPT (eNAMPT) derives, e.g., from adipocytes or vascular cells and supports inflammatory conditions having a cytokine-like function (Romacho et al., 2020). It activates Toll-like-receptor 4 (TLR4; Romacho et al., 2020), promotes B cell maturation or simply fosters pro-inflammatory cytokine production (Dahl et al., 2012) involving not only the innate immune cells neutrophils, monocytes, macrophages, and dendritic cells but also epithelial and endothelial cells (Grahnert et al., 2011). Interestingly, on a vascular level NAMPT has angiogenic potential and enhances endothelial and smooth muscle cell survival (Romacho et al., 2013; Garten et al., 2015; Wang et al., 2016; Chen et al., 2017).

Apparently, increased eNAMPT levels are present in inflammatory and metabolic disorders like insulin resistance, obesity, cardiovascular disease (Sommer et al., 2008), and acute lung injury (Garcia and Moreno Vinasco, 2006). Moreover, eNAMPT appears upregulated in autoimmune diseases, exemplarily, rheumatoid arthritis (Nowell et al., 2006; Otero et al., 2006), psoriasis (Koczan et al., 2005), or inflammatory bowel disease (Moschen et al., 2007), whereas serum levels of eNAMPT could be correlated with the commonly determined inflammation parameters C-reactive protein (CRP) and IL-6 (Oki et al., 2007). In fact, NAMPT or rather visfatin is known as adipocytokine with immunomodulatory and inflammatory function being able to bind to the insulin receptor, to activate leucocytes and cytokine production like IL-1b, TNF-a, and IL-6 in CD14^+^ monocytes and stimulates monocytes leading to upregulated expression of CD54, CD40, and CD80 (Moschen et al., 2007).

It was shown that upregulated NAMPT promotes pulmonary vascular remodeling and proliferation of hPAECs and, thus, can function as biomarker in PAH (Chen et al., 2017). In patients with PAH, levels of NAMPT in the lung and plasma were significantly increased when compared to controls (Chen et al., 2017). Further, PAECs isolated from PAH patients secreted more NAMPT than control cells *in vitro*. Additionally, conditioned medium of these PAECs promoted PASMC proliferation that, in turn, could be arrested when adding NAMPT inhibitor FK866 (Chen et al., 2017). Interestingly, application of FK866 in a rat model of monocrotaline-induced PH starting at the 1st day after monocrotaline injection could prevent PH development and attenuated PH in rats in a Sugen-induced hypoxia model when taking RVSP, RVH, RV contractility index and PA muscularization into account (Chen et al., 2017). These results suggest a direct contribution of upregulated NAMPT expression to PH development, which might clinically function as biomarker and further its inhibition makes up a therapeutic option in PH.

As earlier mentioned, eNAMPT targets Toll-like-receprot-4 (TLR4) a pattern recognition receptor leading to the activation of the NLRP3 inflammasome, which plays a role in inflammatory processes of cardiovascular diseases like atherosclerosis (Luo et al., 2014; Grebe et al., 2018). It was further investigated that TLR4 activation plays a role in eNAMPT related vascular dysfunction (Romacho et al., 2020). In fact, defective endothelium-dependent relaxation in murine mesenteric microvessels induced by eNAMPT could be attenuated by TLR4 inhibitor CLI 095 (Romacho et al., 2020). Cultured endothelial cells with exposure to eNAMPT presented higher expression of NLRP3-inflammasome related proteins and increased levels of p65 NF-κB and IL-1β (Romacho et al., 2020), supporting the fact that NAMPT enhances vascular inflammation *via* TLR4 and, e.g., NAMPT itself or IL-1 blockage may function as therapeutic target in cardiovascular diseases. Moreover, as shown by Gao et al. application of microRNA410 (miR410) mimics in PAECs could prevent the induction of NAMPT and the occurrence of hypoxia induced PH in mice (Gao et al., 2019), stressing the fact that NAMPT can represent a potential target aiming vascular remodeling in PH.

## Innate Immunity

### Increased Numbers of (Inflammatory) Monocytes and Macrophages

Innate immune cells, particularly monocytes and macrophages, play important roles in maintaining tissue homeostasis and orchestrate reparative processes after tissue injury. While macrophage cell populations reside (and reproduce locally) in lungs under physiological conditions, their population increases rapidly in response to injury and pathological stimuli (e.g., PH) by mobilization of monocytes from distal sites (spleen and bone marrow) and their recruitment into specific organs (Florentin et al., 2018). The active recruitment of monocytes to the site of injury and their polarization is orchestrated by the vascular microenvironment and is mainly facilitated by stimuli released from the local tissue milieu, e.g., including damage-associated molecular patterns, chemokines, apoptotic bodies, or fibroblast-derived factors (Amsellem et al., 2017; Lin et al., 2019; Fan et al., 2021; Li et al., 2021).

#### Pulmonary Inflammation

Subforms of PH have been linked to local inflammatory processes in the lung, yet, whether these findings only reflect an association or an actual cause-effect relationship remains a question of controversy. Characteristically, innate immune cells are found in lung vascular lesions and remodeled vessels in patients with idiopathic or secondary forms of PH (Florentin et al., 2018; Ranchoux et al., 2019). This refers specifically to blood-borne monocytes, which have been previously reported to expand in lung explants from iPAH patients, causing increased muscularization of small pulmonary arteries and modulate local immune and cytokine responses (Florentin et al., 2018; Figure 1). CD68^+^ macrophages have been frequently found in advanced obliterative plexiform lesions in experimental and clinical iPAH (Vergadi et al., 2011) and vice versa, macrophage depletion by serial injections of either gadolinium chloride or liposomal clodronate prevented vascular remodeling of small pulmonary arteries in experimentally induced hypoxic PH and portopulmonary hypertension (Thenappan et al., 2011; Tian et al., 2013).

**Figure 3:**
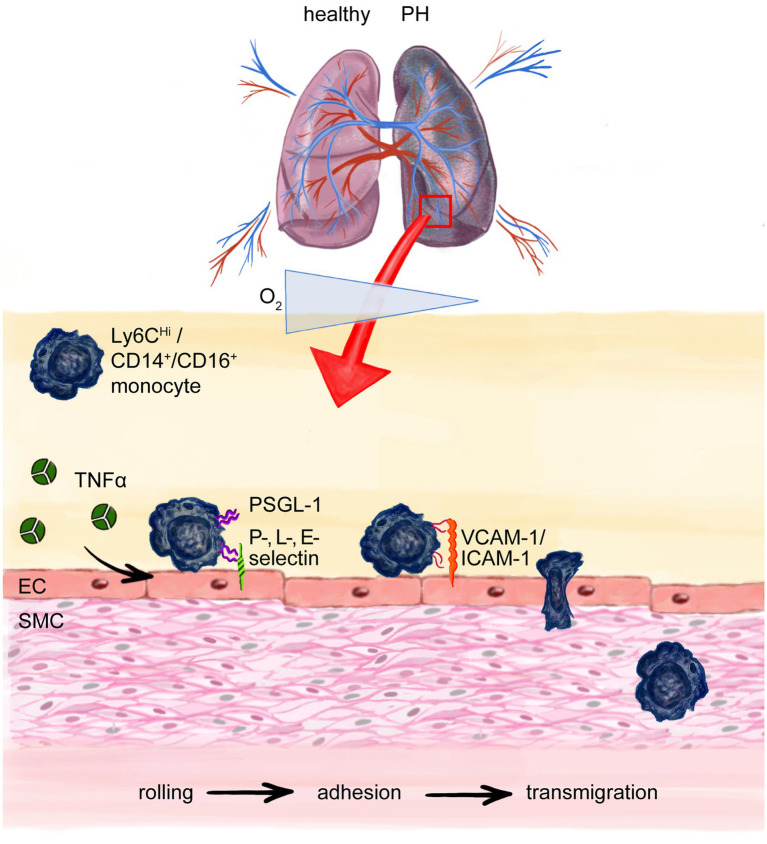
Monocyte transmigration to the site of injury and perivascular inflammation in pulmonary hypertension (PH). Chronic hypoxia in PH recruits monocytes to the lung. Cytokines like Tumor necrosis factor-α (TNF-α) cause upregulation of adhesion molecules on endothelial cells (ECs) e.g. P, L-, E-selectin which interact with P-selectin glycoprotein ligand-1 (PSGL-1) on especially murine pro-inflammatory Ly6C^Hi^ monocytes (correspond to human CD14^+^, CD16^−^ pro-inflammatory monocytes) enabling the process of rolling on and attaching to the endothelium with following integrin mediated adhesion to, e.g., ICAM-1 or VCAM-1 and finally paracellular transmigration. Intercellular adhesion molecule-1 (ICAM-1); vascular cell-adhesion molecule-1 (VCAM-1); smooth muscle cell (SMC).

Chronic hypoxia has been suggested as a key pathogenic driver for the recruitment of monocyte/macrophage lineage precursors (CD45^+^, CD11b^+^, CD14^+^, and CD68^+^) from the bone marrow to the lung, a notion that is supported by the recent finding that monocytes can sense hypoxia, infiltrate pulmonary arteries, and promote vascular remodeling in a murine model of chronic hypoxic PH (Frid et al., 2006; Yu et al., 2020; Figure 3). Traditionally, there are two major subsets of murine monocytes, defined by their expression of the Ly6C antigen and the associated distinct migratory and inflammatory properties: The classical Ly6C^Hi^ monocytes (correspond to CD14^+^, CD16^−^ monocytes in humans) efficiently infiltrate inflammatory sites and are hence often termed as “inflammatory monocytes.” In contrast, nonclassical Ly6C^Lo^ monocytes (correspond to CD14^Lo^, CD16^+^ monocytes in humans) patrol the resting vasculature, repair the endothelium during homeostasis and give rise to anti-inflammatory “alternative” macrophages responsible for inflammation resolution and tissue repair (Gordon, 2003; Gordon and Martinez, 2010; Rahman et al., 2017; Figure 1). Monocytes express high levels of C-C chemokine receptor type 2 (Ccr2) and C-X3-C Motif Chemokine Receptor 1 (Cx_3_cr1), both essential for monocyte retention, survival and, more importantly, egress from the bone marrow and their recruitment to the site of injury. Genetic deficiency of Ccr2 or Cx_3_cr1 resulted in reduced remodeling of the lung vasculature in rodent PAH models (Florentin et al., 2018), pointing toward a role for circulating monocytes in PH-associated vascular remodeling.

#### Sterile Inflammation in Patients With HFpEF

Recently, chronic low-grade sterile inflammation (also called as “meta-inflammation”) has received special attention in the PH-LHD subgroup of HFpEF patients (Paulus and Tschope, 2013). Based on the key role of inflammation-associated non-cardiac comorbidities (e.g., obesity, diabetes, and chronic kidney disease) in HFpEF-pathophysiology, these patients present with higher levels of circulating pro-inflammatory biomarkers including IL-6, TNF-*α* and C-reactive protein (CRP; Abernethy et al., 2018), monocytosis, and pro-inflammatory monocyte differentiation (Glezeva et al., 2015). Antibody-mediated depletion of IL-6 was previously shown to attenuate medial thickening in small pulmonary arterioles, highlighting IL-6 as a critical immune mediator in lung vascular remodeling (Breitling et al., 2017). Such inflammatory signatures are closely associated with echocardiographic parameters of HFpEF and worsened RV function (Sanders-van Wijk et al., 2020), indicating a potential pathogenic and/or aggravating role of systemic inflammation in PH-HFpEF. While cardiac macrophage expansion in humans and mice with diastolic dysfunction results from increased hematopoiesis in bone marrow and spleen (Hulsmans et al., 2018), similar data regarding the role of innate immune cells in extracardiac organs like the lung is missing so far. Although it seems almost self-evident that systemic meta-inflammation in the HFpEF syndrome affects a multitude of organs it remains to be shown how precisely enhanced hematopoiesis and resulting monocytosis could contribute to the lung vascular maladaptation in HFpEF patients.

#### Monocyte Recruitment to the Site of Inflammation and Injury

On a mechanistic level, monocyte recruitment to the site of injury and their extravasation are crucial events in early vascular inflammation and require close interaction of ECs and monocytes (Gerhardt and Ley, 2015). When chronically activated, the endothelium upregulates the expression of adhesion molecules, secretes growth factors and cytokines that in turn affect EC and smooth muscle cell (SMC) proliferation, apoptosis, and favor the transcellular migration of inflammatory cells, ultimately resulting in EC dysfunction and vascular remodeling (Breitling et al., 2015). Monocyte trafficking across the vessel wall is regarded as a multistep process, tightly regulated and involving the stages of rolling, adhesion and transmigration (Ley et al., 2007; Figure 3). Initially, pro-inflammatory cytokines like TNF-α secreted by leukocytes, transiently activate the endothelium and cause rapid expression of adhesion molecules on the luminal surface of ECs (Imhof and Aurrand-Lions, 2004). Adhesion molecules engage with, e.g., O-glycosylated carbohydrate ligands displayed on P-selectin glycoprotein ligand-1 (PSGL-1), which is expressed on all monocytes (Gerhardt and Ley, 2015). Functionally, this allows monocytes to slowly roll on and attach to the endothelium, withstanding the shear stress of the blood flow in the vasculature. PSGL-1 is expressed at significantly higher levels by Ly6C^Hi^ monocytes than by reparative Ly6C^Lo^ monocytes, which makes Ly6C^Hi^ monocytes more likely for adhesion and subsequent migration (Figure 3). In line with this notion, TNF-α signaling drives PAH development by suppressing BMPR2 signaling (Hurst et al., 2017). Moreover, mice overexpressing TNF-α develop severe PH, chronic inflammation and RV hypertrophy (Fujita et al., 2001, 2002). Mechanistically, TNF-α signaling increases endothelial-leukocyte cell interaction, by increasing the expression of cell adhesion molecules on ECs, paving the way for leukocyte influx into the injured vasculature (Min et al., 2005). Of note, serum TNF-α is chronically elevated in heart failure patients (Levine et al., 1990; Ridker et al., 2000), pointing toward a crucial role of pro-inflammatory cytokines in general and TNF-α in particular in the onset and progression of secondary vascular inflammation and PH development.

### Impaired Function of Natural Killer Cells

Natural killer (NK) cells are lymphoid cells that form 5%–20% of circulating lymphocytes in humans (Abel et al., 2018). They are part of innate immunity and possess the ability to eliminate virally infected cells or target transformed cells, and, therefore, are considered cytotoxic (Vivier et al., 2018).

#### Angiogenesis and Vascular Remodeling

It is appreciated that NK cells comprise similar functional properties in the innate immune response like cytotoxic T-cells in adaptive immunity (Abel et al., 2018). They can be found in the blood, but also in various organs, i.e., bone marrow, lymphoid tissue, lung, and liver (Pfefferle et al., 2020). NK cells express various tumor necrosis factors such as FASL and TRAIL and produce cytokines, e.g., their principle cytokine IFN-γ, but also TNF-α, IL-10 and the chemokines CCL3, CCL4, CCL5, and XCL1. Further, they interact with other immune cells, especially DCs, macrophages and T-cells in terms of, e.g., supporting DC maturation and suppressing or empowering macrophage and T cell responses *via* IFN-γ or IL-10 secretion (Vivier et al., 2011; Figure 1). Extending beyond these functions, NK cells may regulate vascular remodeling, and, thus, are of great interest referring to the pathophysiological understanding of PH and their potentially protective property (Ormiston et al., 2012). While it is known that decidual NK cells are crucial to regulate angiogenesis during pregnancy (Hanna et al., 2006), also in mice with NK cell depletion through targeting with anti-NK1.1 antibody and in NK cell deficient transgenic mice arteriogenesis was impaired (van Weel et al., 2007).

#### NK Cell Deficiency and Dysfunction in PH

On the contrary, NK cell deficiency combined with depletion of CD8^+^ T-lymphocytes rather comes along with a short-term survival rate. In their study, Edwards et al. included patients with IPAH and connective tissue disease-associated PAH (CTD-PAH) and correlated lymphocyte profiles with survival rate within the first 3 years, revealing that low levels of NK cells and CD8^+^ T-lymphocytes were associated with increased risk of death, particularly in patients with CTD-PAH (Table 1). However, due to the small number of patients further studies need to verify these results and ponder on the question whether NK cell depletion, which is usually common in severe viral infections or cancer (Cooper et al., 2001), represents consequence or causative agent in PH (Edwards et al., 2013; Table 1). In line with these findings, Ormiston et al. found decreased CD56^dim^/CD16^+^ NK cells in PAH patients (Ormiston et al., 2012). Further isolation of NK cells revealed a functional impairment illustrated by decreased expression of the cytotoxicity receptor NKp46 and inhibitory killer immunoglobulin-like receptor 3DL1 (KIR3DL1) with consequent decreased affinity to degranulate and lessened cytokine secretion measured by macrophage inflammatory protein-1β production (Ormiston et al., 2012; Table 1). Moreover, elevated transforming growth factor-β (TGF-β) signaling and enhanced expression of matrix metalloprotease-9 (MMP-9) was present in patients with PAH (Ormiston et al., 2012). As MMP production of NK cells during pregnancy affects smooth muscle cell apoptosis and demuscularization of arteries, one can suggest a directly protective impact of circulating NK cells on vascular remodeling (Ormiston et al., 2012; Figure 1). Additionally, in mouse models of NK deficiency, IL-23 elevation and spontaneous development of PH takes place, expressed by increased right ventricular systolic pressure, right ventricular hypertrophy, and muscularization of the pulmonary arteries (Ratsep et al., 2018).

#### Protective Activity of NK Cells in PH

Of interest, IL-23 promotes the differentiation of T_H_17 cells to pathogenic regulatory T-cells in autoimmune disease (Singh et al., 2019). The exact role of IL-23, the underlying mechanisms of its increased production and the potential interplay of NK and T_H_17 cells in this process are yet to be determined in further studies. Nonetheless, these results further suggest a naturally beneficial effect of NK cells on the pathophysiology of PH (Ratsep et al., 2018; Table 1).

The potential protective role of NK cells in PH might also extend to right ventricular function. It was observed that Fischer rats exposed to a single injection of VEGFR2 antagonist, SU5416 (SU) and following exposure to chronic hypoxia (SU-Hx model of PAH) suffered from high mortality risk by 7 weeks compared to Sprague Dawley rats (Suen et al., 2019). SU-Hx Fischer rats, that depict RV dysfunction under chronic hypoxia, show downregulated NK-mediated genes in transcriptomic analyses of the right ventricle. This strain-dependent malfunctioning RV adaption can function as a model for RV dysfunction in patients with PAH (Suen et al., 2019; Table 1). Taking these findings into account, it seems rather tempting to speculate whether restoring NK cell function might reverse RV dysfunction in PH.

Interestingly, dysregulated NK cells have been observed also in inflammatory heart diseases, involving myocarditis, myocardial infarction, and cardiac fibrosis (Ong et al., 2017). Patients with ischemic heart disease show decreased number of CD56^bright^ and CD56^dim^ circulating NK cells with limited cytotoxic ability and IFN-γ production similar to the findings in PH (Hak et al., 2007; Table 1). Moreover, patients with myocarditis and dilated cardiomyopathy develop decreased levels of NK cells. Equally to their role in PH, whereas their character of controlling inflammation and, thus, disease severity seems well documented, the exact mechanisms and resulting therapeutic targets remain essential gaps of research to be filled in (Ong et al., 2017).

### Dendritic Cells: Contributors to Altered Immune Responses

Autoimmune diseases, such as SSc or systemic lupus erythematosus (SLE) are commonly accompanied by PAH (Johnson and Granton, 2011), which indicates that the activation of innate and adaptive immune cells plays a fundamental role in PH. In fact, elevated blood pressure and the release of pro-inflammatory mediators in the pulmonary (micro-) circulation induce the formation of bronchus-associated lymphoid tissues (BALTs) in several forms of PH.

#### DC Accumulation in Tertiary Lymphoid Organs

**Figure 4:**
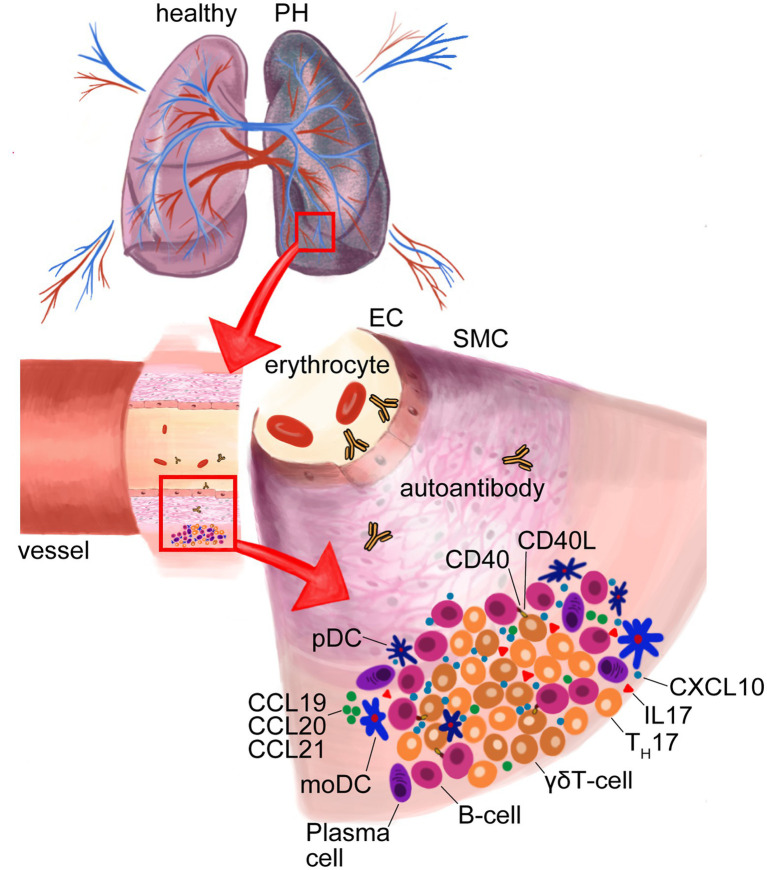
Formation of tertiary lymphoid organs (TLOs) in lungs of pulmonary hypertension (PH). Dendritic cell (DC)-attracting chemokines C-C-chemokine-ligand-20 (CCL20), CCL19 and CCL21 in TLOs cause DC accumulation. Activated plasmacytoid (pDCs) secrete C-X-C motif chemokine ligand 10 (CXCL10) that recruits γδT-cells, which, in turn, activate B-cells *via* CD40-ligand (CD40L) and, thus, support the formation of autoantibodies by plasma cells. MoDCs effect T_H_17 differentiation that produce the proinflammatory interleukin-17 (IL-17). Endothelial cell (EC); smooth muscle cell (SMC).

In close proximity to pulmonary blood vessels, these tertiary lymphoid organs (TLOs) are highly organized structures that show comparable organization to lymph nodes, with distinct T- and B-cell zones, high-endothelial venules, lymphatic vasculature and, importantly, monocytes and several DC subsets (Figure 4). The localization of these immune-active structures adjacent to remodeled pulmonary arteries of iPAH patients suggests an impact of immune cells on vascular remodeling (Perros et al., 2012). Activation of the lymphotoxin (LT)βR signaling is pivotal for the formation and integrity of TLOs in chronic inflammatory disease, which triggers, e.g., the release of lypmhorganogenic chemokines CXCL13 and CCL21 in medial SMCs and promotes the integrity of high-endothelial venules (Grabner et al., 2009; Lotzer et al., 2010). Together this points toward the importance of LT*α*_1_β_2_-expressing DCs in TLO organization and beyond that in PAH pathophysiology. The increase of DC-attracting chemokines CCL20, CCL19, and CCL21 in TLOs (Fleige et al., 2018) may explain a decrease in blood DC populations (Wang et al., 2009; Hautefort et al., 2015; Rohm et al., 2019) as well as centralization of DCs subtypes in TLOs around remodeled pulmonary vessels, which was observed in PAH patients (Perros et al., 2007, 2012; Savai et al., 2012; Marsh et al., 2018; Figure 4).

#### CD4^+^ T_H_-Cell Responses and Tolerance of T- and B-Cells

DCs are phagocytic and, through the expression of the major histocompatibility complex (MHC) class-I and -II molecules, professional antigen presenting cells that have a prominent role in linking innate and adaptive immunity by initiating tolerance and memory in B- and T-cells. By the expression of a multitude of co-stimulatory molecules and the release of pro-inflammatory mediators they shape the T-effector cell populations by polarizing, e.g., T_H_1, T_H_2, and T_H_17 cells in inflammatory processes (Figure 1). The major phenotypically and functionally distinct DC subtypes are divided in conventional DCs (cDCs), plasmacytoid DCs (pDCs), Langerhans cells and the monocyte-derived DCs (moDCs). cDCs can be classified into type-1 cDCs (cDC1) and type-2 cDCs (cDC2), which both share the ability to drive CD4^+^ T_H_-cell responses by antigen presentation through MHC-II. However, cDC1s release IL-12 and play an important role in facilitating tolerance against exogenous (self-) antigens that they cross-present *via* MHC-I to CD8^+^ cytotoxic T-cells, while cDC2s have the distinct ability to trigger T_H_1, T_H_2, T_H_17, T_reg_, or T-follicular helper cell (T_FH_) polarization. Therefore, cDC2s can induce T-cell help to B-cells, which shapes memory formation and may lead to T- and B-cell autoimmunity, respectively. pDCs are well-known to produce type-I IFN and, therefore, specifically contribute to anti-viral immunity. MoDCs originate from blood monocytes, develop upon infection and enter tissues from the blood stream to further exacerbate inflammation through the release of pro-inflammatory mediators such as IL-1β, IL-12, IL-23, TNF-α and iNOS (Figure 1).

#### MoDCs and IL-6 Production

Alterations, impaired migration and increased activation of DC subset in blood and lung have been recognized as underlying aggravating factors of PAH. Through the pro-inflammatory state of patients with iPAH, disease-associated moDCs are capable to efficiently induce CD4^+^ T-cell proliferation and acquire a reduced capacity to induce T_H_2 responses in mixed leukocyte reactions *in vitro* (Hautefort et al., 2015). Importantly, patient moDCs are able to induce T_H_17 differentiation and, therefore, shape an inflammatory environment in pulmonary TLOs fostering autoimmunity (Hautefort et al., 2015; Figure 4). Interestingly, moDCs in the blood circulation of SSc patients show TLR2- and TLR4-dependent production of IL-6, IL-10, and TNF-α (van Bon et al., 2010; Carvalheiro et al., 2018). In the immunopathology of PAH, these cytokines receive specific attention as IL-6 and IL-10 correlate with disease-associated mortality (Soon et al., 2010) and are specifically high in connective tissue disease (CTD)-associated PAH (Carvalheiro et al., 2018). Furthermore, overexpression of IL-6 in mice causes elevated pulmonary vascular remodeling and PAH (Steiner et al., 2009), while IL-6 deficient mice are not susceptible for PH (Savale et al., 2009) and ectopic upregulation of membrane-bound IL-6 receptor (IL-6R) on PA-SMCs rodent models of PH fosters vascular remodeling that can be attenuated with IL6R/sIL6R antagonist ERBF and an IL-6R-neutralizing antibody (Tamura et al., 2018; Figure 1).

#### cDC1s and cDC2s

Since it has been uncertain if changes in DCs immunity represents reason or cause of pulmonary vasculature remodeling leading to hypertension, the DC activation pathway in the onset and progression of PH is a matter of consideration. A central role in the immune modulation of PH is attributed to the key regulatory ubiquitin-binding protein A20 (encoded by TNFAIP3), as polymorphisms in the TNFAIP3 locus are strongly associated with the development of PAH (Dieude et al., 2010; Ma and Malynn, 2012). Koudstaal et al. recently aimed to elucidate whether constitutive activation of cDCs in mice would result in the development of PH. To this end, they conditionally deleted TNFAIP3 in cDC2s and assessed RV systolic pressure, RV hypertrophy, pulmonary vascular remodeling, and IL-6 production (Koudstaal et al., 2020). Strikingly, all these characteristic parameters of experimental PH were increased in Tnfaip3^DNGR1^-KO mice and could be attenuated by administration of neutralizing anti-IL-6 antibody (Koudstaal et al., 2020). Encouraging the idea that DCs are of outmost importance regarding the maintenance of immune surveillance and lung homeostasis, it was shown that activated DCs deficient for CCR7 promote lung inflammation and PH in mice (Larsen et al., 2011; Fleige et al., 2018). As DCs sense and follow CCL19 and CCL21 gradients expressed by lymphatic ECs through CCR7 in order to migrate to draining lymph nodes, it is likely that pulmonary DC retention contributes to PH. This hypothesis is supported by the observation that arterial DC accumulation precedes muscularization in monocrotaline-treated rats (Perros et al., 2007).

The persistent pulmonary inflammation is the underlying trigger for acquired autoimmune features of PAH (Perros et al., 2007; Colvin et al., 2013).

#### pDCs and γδT-Cells

During inflammation, pDCs are activated and promote adaptive immune responses in peripheral tissues. IPAH patients show elevated moDCs concentrations in close vicinity to pulmonary arteries and, additionally, increased pDCs numbers predominantly localized in the alveolar space in proximity to the microcirculation (Marsh et al., 2018). Further, activated pDCs show augmented production of the IFN-inducible chemokine CXCL10 (Liu et al., 2013). CXCL10 is associated with PAH in SSc patients and causes RV dysfunction (Eloranta et al., 2010; Yang et al., 2014; Figure 1). Intriguingly, CXCL10 induces adaptive immune responses by recruiting CXCR3+ cells, including γδT-cells. γδT-cells are unconventional T-cells interacting with other innate and adaptive immune cells and can enhance or suppress inflammation. It was previously reported that γδT-cells release insulin-like growth factor (IGF) -1—a factor that stimulate pulmonary smooth muscle cell proliferation (Dempsey et al., 1994). Importantly, a strong increase in the abundance of γδT-cells in iPAH patients was observed (Marsh et al., 2018). Since the role of γδT-cells in autoimmune disease is to help B-cells to produce autoantibodies through costimulatory molecules like CD40L, inducible T-cell co-stimulator (ICOS), and CD28 and the release of pro-inflammatory cytokines (Rampoldi et al., 2020), it is tempting to assume that pDCs provide a link in the recruitment and activation of γδT-cells that, in turn, shape B-cell autoimmunity of IPAH (Figure 4).

## Adaptive Immunity

### Affected B-Cell Development and Production of Autoantibodies

PH causes sterile injury-associated cell death and, in turn, passive release of biologically active pro-inflammatory lipid mediators, cytokines, and chemokines. Therefore, a growing body of evidence shows that the different forms of PH are accompanied by sterile inflammation and recruitment of not only innate but also adaptive immune cells, which form characteristic TLOs in PH lungs (Figure 4). In TLOs, B-cell follicles and T-cell rich areas allow the interaction between lymphocytes and (auto-) antigen presenting DCs on a matrix formed by a network of mesenchymal cells, while high-endothelial venules allow the extravasation of naïve transitional lymphocytes. In TLOs, as in secondary lymphoid organs, B-cells are negatively selected according to their binding affinity of self-antigen with the B-cell receptor (BCR), or get the chance to undergo affinity maturation by receptor editing. Autoimmunity develops if these negative selection processes are impaired and the maturation of activated B-cells results in plasma cell differentiation and autoantibody production (Figure 4).

#### Activation of B-Cells and Plasma Cells

It is a long-standing observation that hypertensive patients show elevated serum levels of IgM, IgG or IgA (Ebringer and Doyle, 1970; Gudbrandsson et al., 1981; Suryaprabha et al., 1984; Hilme et al., 1989), but their causative role in hypertension has been shown just recently in Angiotensin-II-treated mouse models (Chan et al., 2015). Experimental hypertension was associated with strong increases in splenic plasma cells and circulating IgG levels, accompanied by IgG deposits in the aortic adventitia promoting vascular remodeling (Chan et al., 2015; Figure 1). Importantly, B-cell activating factor receptor (BAFFr)-deficient mice, which are impaired in mature B-cell and plasma cell differentiation and show strong IgG reduction, only develop hypertension in response to Angiotensin-II when receiving adoptive B-cell transfer (Chan et al., 2015). Further, Angiotensin-II-induced hypertension could be reduced by anti-CD20 B-cell depletion (Chan et al., 2015). These data underline the role of B-cell immunity on vascular remodeling in hypertension, and suggest that activated B- and plasma cell responses bear the potential to aggravate PH.

#### The Mast Cell/B-Cell Axis in PH

Notably, genetic deficiencies or pharmacological interventions leading to inhibition or depletion of mast cells (Dahal et al., 2011; Hoffmann et al., 2011) or B-cells (Mizuno et al., 2012; Breitling et al., 2017) confer protection from PH in various preclinical disease models, including those of PH-LHD. In particular, mast cell-derived IL-6 received specific attention as a critical link driving B-cell-mediated adaptive autoimmune reactions accompanied by the formation of TLOs in PH lungs (Breitling et al., 2017). The soluble cytokine IL-6 plays an important role in acquired immune responses by stimulating terminal B-cell differentiation and autoantibody production, as well as effector T-cell development (Hirano, 1992; Akira et al., 1993). Because of the regulatory effects of IL-6 on B-cell differentiation, the immunoglobulin repertoire and the control of autoimmune checkpoints, a mast cell dependent control of IL-6 release, which regulates the lung B-cell pool in PH was investigated (Breitling et al., 2017). Rats with monocrotaline-induced PH exhibit, besides mast cell accumulation and elevated levels of IL-6, a prominent formation of TLOs with highly proliferative B-cell follicles and increased depositions of autoantibodies along the lung microvascular walls, suggesting a potential contributory role of B-cells in the development and progression of PH (Breitling et al., 2017). This notion is supported by studies that described a high prevalence for PAH when autoantibody deposition in pulmonary artery walls was detected in patients with connective tissue diseases, i.e., systemic sclerosis (Chung et al., 2010; Chaisson and Hassoun, 2013; SSc-PAH) or Sjögren’s syndrome (Kobak et al., 2014).

#### Development of and Protection Against Autoimmunity

During their development, B-cells pass multiple checkpoints in the bone marrow and in the periphery where the BCR is probed for its autoreactivity. B-cell tolerance mechanisms, such as receptor editing by somatic hypermutation and affinity maturation, warrant the fate of developing B-cells and protect them from clonal deletion. The B-cell follicles in pulmonary TLOs exhibit highly active germinal centers where the modification of the variable and constant regions of the B-cell immunoglobulin genes leads to the maturation of B-cells. Subsequently, impaired negative BCR selection causes enrichment in autoreactive effector cells, which could contribute to disease pathogenesis locally. Indeed, anti-phospholipid (Karmochkine et al., 1996), anti-nuclear (Rich et al., 1986), anti-fibroblast (Tamby et al., 2006), anti-EC (Dib et al., 2012), and anti-fibrillin (Morse et al., 2000) antibodies were described to be involved in the initiation and/or progression of iPAH. Circulating anti-endothelin receptor type A and anti-angiotensin-II receptor type 1 autoantibodies were indeed identified as predictive and prognostic biomarkers in SSc-PAH (Becker et al., 2014). Interestingly, deficiency of the P-, E- and L-selectin binding molecule PSGL-1 leads to an SSc-like syndrome and SSc-associated PH in female mice (Gonzalez-Tajuelo et al., 2020). PSGL-1 is specifically expressed in mature innate-like B1a and B1b, conventional B2 B-cells as well as in plasma cells (Gonzalez-Tajuelo et al., 2020). Remarkably, PSGL-1 expressing B-cells are activated IL-10-producing regulatory B effector cells (B_reg_) owing a protective function in the development of autoimmune disease. In fact, B_reg_ cells are significantly reduced in SSc patients with PAH but not in patients with interstitial lung disease (Ricard et al., 2021). Of note, it appears that the frequency of circulating T_FH_ cells negatively correlates with the B_reg_ proportion in the blood of SSc-PAH patients, suggesting regulation of T_FH_ populations and PAH prevalence by B_reg_-cells (Ricard et al., 2021). Beyond its functional relevance in animal models, a reduction of PSGL-1 in mature B-cells of iPAH patients in comparison to healthy controls was detected (Gonzalez-Tajuelo et al., 2020), indicating that PSGL-1 dysregulation on B- and plasma cells in iPAH may lead to impaired peripheral tolerance and negative selection of autoreactive B-cells, thereby promoting the development of PH (Figure 1).

Despite the emerging role of autoimmunity in PH, the humoral immune basis and the analysis of the immunoglobulin repertoire in PH has only recently been addressed and is limited to one study in SSc-PAH patients who only form a small proportion of all PH cases (de Bourcy et al., 2017). Rats with LV heart failure following supracoronary aortic banding show elevated total IgG levels in plasma (Breitling et al., 2017) and are protected from the development of severe experimental PH-LHD when mast cells are ablated (Hoffmann et al., 2011). Strikingly, the mast cell stabilizer Ketotifen protected rats with left heart failure from reactive PH, in that it largely normalized mPAP and pulmonary vascular resistance (PVR; Hoffmann et al., 2011). It appears plausible that the effect of Ketotifen expands on the mast cell/B-cell axis, but further investigation will have to probe this notion. Therefore, and together with the recognition of the B-cell autoimmune component in several forms of PAH, an overarching importance of B-cell mediated autoimmunity in PH emerges. Therefore, the efficacy of B-cell depletion therapy in SSc-PAH patients has been recently assessed in a multicenter, double-blinded, randomized, placebo-controlled proof-of-concept trial (Zamanian et al., 2021). Treatment with the chimeric monoclonal anti-CD20 antibody rituximab did not reach its endpoints in improved exercise tolerance and improved pulmonary vascular resistance in patients with SSc-PAH (Zamanian et al., 2021). However, in a *post hoc* analyses of the rituximab-treated group, patients bearing increases in the biomarkers rheumatoid factor, IL-2 and IL-17 showed significant lower pulmonary vascular resistance in comparison to patients that did not have these alterations and received antibody therapy, and, therefore, raise awareness for patient selection for B-cell depletion therapies in SSc-PAH (Zamanian et al., 2021; Zhang and Michelakis, 2021). However, the dysregulated mechanisms leading to the maturation of autoreactive B-cells in PH in general and in PH-LHD in particular are poorly understood. The in-depth dissection of B-cell and plasma cell mediated autoimmunity in PH-LHD deserves further attention in order to foster our understanding of basic disease mechanisms and to advance in the identification and testing of novel therapeutic targets.

### Imbalance of T-Cell Subsets

The diversity of the T-cell compartment in various organs encompasses a great entity of CD4^+^ and CD8^+^ T lymphocytes varying in differentiation, migration capacities, residence, and characteristically in their role in immunoregulation with regard to cytokine production, their abilities in modulating inflammation and effector function (Dong, 2021). While CD8^+^ T-cells are considered cytotoxic (cytotoxic T lymphocytes, CTL) and are fundamental for the clearance of intracellular pathogens, CD4^+^ T-cells make up a heterogenous group of T_H_ cells, which includes but is not limited to T_H_1, T_H_2, T_H_9, T_H_17, and T_FH_ cells that can be distinguished mainly by their cytokine expression profiles. Additionally, there are T_reg_-cells that are important for maintaining immune homeostasis by regulation of immune responses, and, therefore, naturally prevent autoimmune diseases (Beissert et al., 2006; Dong, 2021). Recently, alterations in the homeostasis of diverse T-cell subsets have aroused substantial interest as contributors in the pathogenesis of cardiovascular disease in general and even of PH in particular, as intriguingly demonstrated for example in a preclinical study, in which athymic mice with subsequent T-cell deficiency developed PAH (Tamosiuniene et al., 2011).

#### Cytotoxic T Lymphocytes

While deficiency in T-cells functions as marker in chronic viral infections like viral hepatitis and human immunodeficiency virus (HIV) infection as well as in cancer (Wherry, 2011), combined CTL and NK cell depletion was associated with elevated mortality in iPAH and CTD-PAH patients within 10 months of time (Edwards et al., 2013). Independently, CTL depletion was further accompanied by inferior 6 min walking test results and heightened grading in NYHA classification, emphasizing a link between CTL depletion and worsened clinical outcome in patients with PH (Edwards et al., 2013). Indeed, decreased CTL levels in iPAH patients have already been described by Ulrich et al. but have not been linked to clinical parameters and disease outcome thus far (Ulrich et al., 2008; Li et al., 2017).

#### T_regs_

Restriction and downregulation of immune responses is fundamental to control inflammatory conditions and to sustain self-tolerance and is thus of particular importance in the prevention of autoinflammation and autoimmunity. To date, T_regs_ which secret the anti-inflammatory cytokine IL-10 (Martini et al., 2020; Tian et al., 2021) have been studied in the context of various cardiovascular diseases like atherosclerosis (Meng et al., 2016) and also PH where they have beneficial effect in terms of disease progression, implying that T_reg_ deficiency or malfunction might trigger disease aggravation (Martini et al., 2020; Tian et al., 2021; Figure 1).

In addition, T_regs_ prohibit antigen presentation and DC maturation through IL-10 secretion and a subsequent reduction in MHC-II expression (Sziksz et al., 2015), while inhibiting T-cell proliferation and differentiation by granzyme B and CD73 expression (Qiu et al., 2019). In PAH T_regs_ confer regulatory functions in pulmonary arterial EC (PAEC) injury and pathologic PASMC proliferation and apoptosis (Qiu et al., 2019). Of note, T_regs_ can downregulate PAEC proliferation through anti-inflammatory cytokine release and hence inflammatory response in general (Qiu et al., 2019). Transfer of CD4^+^ CD25^+^ T_regs_ isolated from human peripheral blood mononuclear cells (PBMCs) to mice with hypoxia-induced PH improved the PH phenotype and vascular remodeling in these animals (Chu et al., 2015). mRNA and protein expression analyses indicated a significant reduction of MCP-1, IL-1β and IL-6 in animals with extrinsic T_regs_ while the ani-inflammatory cytokine IL-10 appeared to be upregulated. Further analyses included the assessment of T_reg_ dependent effects on the metabolic activity of human pulmonary artery smooth muscle cells (hPASMCs) using a cell counting assay. Interestingly, the proliferation of hPASMCs was inhibited encouraging the assumption that T_regs_ embody disease-limiting features in PH (Chu et al., 2015).

In T-cell deficient rats immune-reconstitution with CD4^+^ CD25^+^ or CD25^−^ T_regs_ prior to SU5416 administration (HySU) could reduce endothelial apoptosis, protected against pathological RV adaption and caused upregulation of vascular BMPR2 expression (Tamosiuniene et al., 2011). Nevertheless, a causal relationship between T_regs_ and BMPR2 expression has not been revealed so far. Investigating the direct mechanisms of T_reg_-induced endothelial BMPR2 expression is hence of great interest to understand the contribution of T_reg_-dependent preservation and repair of the pulmonary vasculature and the potential of Treg immunotherapy in human PH and its proper time point of application.

#### T_H_2 Cells and γδT-Cells

Interestingly, apart from the aforementioned subsets, perivascular deposition of CD4^+^ T-cells were observed in iPAH patients (Savai et al., 2012). This subset of T_H_ cells produces IL-6, IL-2, IL-21, IFN-γ, and TNF-*α* and is generally associated with the promotion of inflammation in PAH (Qiu et al., 2019; Figure 1).

More recently, Chen et al. demonstrated that the chemoattractant receptor homologous molecule expressed on T_H_2 cell (CRTH2) expression in CD4^+^ T-cells was elevated in patients with PAH and rodent PAH models (Chen et al., 2018). CRTH2 is a chemokine receptor for, amongst others, prostaglandin D_2_ (PGD_2_) and induces chemotaxis and IL-4, IL-5 and IL-13 release in T_H_2 cells (Xue et al., 2005). CRTH2-deficient mice in an HySU model presented an improved PH phenotype based on reduced right ventricular systolic pressure (RVSP), Fulton’s index, vascular wall thickness and decreased CD4^+^ T-cell perivascular deposition and IL-4 and IL-13 levels when compared to HySU-treated wild-type mice (Chen et al., 2018).

Concluding, even though a direct lineage-specific link between CRTH2 and T_H_2 cells could not be depicted, these results suggest a contribution of T_H_2 cells to the pathogenesis of PH. Intriguingly, activated T_H_2 cells can induce IgE production of B-cells and consequent activation of mast cells and eosinophils through IL-4 (Corry and Kheradmand, 1999). As noted in earlier chapters, the involvement of mast cells in perivascular remodeling and subsequent disease development in iPAH patients and experimental PAH could be shown already (Dahal et al., 2011; Bartelds et al., 2012). In line with the disease-promoting role of T_H_2 cells, the subset of γδT-cells was also increased in patients with iPAH (Marsh et al., 2018), being responsible for tissue repair, induction of inflammation and recruitment of leukocytes (Fay et al., 2016).

#### T_H_17 Cells

Beyond the previously described T-cell subsets, T_H_17 cells are known to be involved in autoimmune, airway and cardiovascular diseases by promoting neutrophil mobilization and releasing pro-inflammatory cytokines like IL-6, IL-8, IL-11, and IL-17 and growth factors GM-CSF and VEGF with aggravating effect on airway remodeling and tissue damage (Linden, 2006; Cheng et al., 2008; Maston et al., 2017; Figure 1).

Moreover, human rho-associated kinase (ROCK) signaling in T-cells modulating immune response and cytokine release is of recent interest (Zanin-Zhorov and Waksal, 2015). The serine–threonine kinases ROCK1 and ROCK2 are activated by Rho GTPase and cause downstream phosphorylation of cellular targets (Riento and Ridley, 2003). Actually, recent studies revealed that in psoriasis, a common autoimmune disorder of the skin, the ROCK2 inhibitor KD025 downregulated IL-17 levels, and thus T_H_17 immune response, with additional upregulation of IL-10 and subsequent clinical improvement after 12 weeks of treatment (Zanin-Zhorov et al., 2017). Upregulation of ROCK signaling is also relevant in other autoimmune diseases such as rheumatoid arthritis (He et al., 2008) and SLE (Isgro et al., 2013; Tengesdal et al., 2018), suggesting ROCK as potential therapeutic target in diseases with underlying T-cell dysregulation. Since IL-17 levels are elevated in PAH patients signifying an intensified T_H_17 immune response (Hautefort et al., 2015), inhibition of ROCK signaling in PH seems worth considering. Li et al. studied alterations of T_H_17 and T_reg_ homeostasis when rats were treated with RhoA-ROCK pathway activator lysophosphatidic acid (LPA) or the respective nonselective ROCK-inhibitor fasudil (FSD) in a chronic hypoxia model (Li et al., 2018). Increased expression of phosphorylated signal transducer and activator or transcription 3 (p-STAT3) and reduced expression of p-STAT5 in peripheral blood and spleen by LPA was accompanied with an increase in IL-17 levels and reduction of IL-10 levels. Interestingly, the application of FSD showed opposing effects (Li et al., 2018). Thus, an activation of the RhoA-ROCK pathway *via* LPA exacerbated vascular remodeling, while an inhibition *via* FSD hypoxic rats showed an increase in T_regs_ and reduced vascular remodeling, implying that modulating the RhoA-ROCK pathway affect T_H_17 and T_reg_ homeostasis (Li et al., 2018).

Additional studies showed that RAG^−/−^ mice lacking B and T lymphocytes, which were exposed to chronic hypoxia, had reduced RVSP and pulmonary arterial remodeling as compared to WT mice after exposed to chronic hypoxia (Maston et al., 2017). These mice then received adoptive CD4^+^ T-cell transfer causing a shift to the PH phenotype. Of interest, RAG^−/−^ mice that only received T_H_17 cells seemed to develop PH even without induction of hypoxia (Maston et al., 2017). Interference of T_H_17 development and function by inhibiting the retinoid-related orphan receptor RORγt, which is essential for T_H_17 differentiation (Solt et al., 2011), with SR1001, reduced RVSP in mice with hypoxia-induced PH (Maston et al., 2017). These results indicate a divers and complex contribution of CD4^+^ T-cells to the pathophysiology of PH. Promoting T_reg_ function as well as suppressing T_H_17-driven inflammation and vascular remodeling might make up potential therapeutic targets to be further investigated in upcoming studies.

#### Galectins

In the context of T-cell immunity, emerging evidence suggests that β-galactoside-binding lectins, namely Galectins, possess crucial functions in immune homeostasis and also in the pathophysiology of cardiovascular diseases (van der Hoeven et al., 2016) by controlling innate and adaptive immunity, namely by triggering the production of pro-inflammatory cytokines, T_reg_ promotion and T-cell differentiation (Ilarregui et al., 2005).

In patients with SSc, Galectins gained special interest due to their multifarious involvement in inflammation, immune response, vascular remodeling (Johannes et al., 2018) and some subsets of Galectins appeared to be decreased in patients with systemic sclerosis (Taniguchi et al., 2012; Yanaba et al., 2016; Saigusa et al., 2019) and in patients with PAH due to congenital heart disease (PAH-CHD; Shen et al., 2019). Galectin-10 (Gal-10) is mainly expressed in eosinophils, basophils, and T_regs_ and functions as biomarker in of airway inflammation in eosinophilic asthma (Johannes et al., 2018). Actually, Gal-10 suppresses T-cell proliferation through cell–cell interaction with eosinophils (Lingblom et al., 2017), raising the question if eosinophils possess a regulatory function in SSc and subsequent PH. Of note, Gal-10 serum levels of SSc patients correlated inversely with RVSP data, supporting the idea of protective function of T_regs_ and potential impaired function of eosinophils in terms of developing PH (Awaji et al., 2021). Further, Galectin-1 (Gal-1) is exemplarily expressed in cardiomyocytes, ECs, VSMCs, activated macrophages and activated T-cells (Fuertes et al., 2004; Cerliani et al., 2011) and appears upregulated in acute myocardial infarction (Al-Salam and Hashmi, 2014) and in chronic hypoxia model of PH (Case et al., 2007). Of note, Gal-1 plays a role in the apoptosis of CD8^+^, T_H_1, and T_H_17 cells (Toscano et al., 2007), while increased levels of T_H_1 and T_H_17 is typically present in acute coronary syndrome (Cheng et al., 2005; Seropian et al., 2018). These results depict a contribution of Gal-1 to the pathophysiology of impaired T-cell function in multiple cardiovascular diseases while a deeper mechanistic understanding remains elusive.

Taken together, the mechanistic role of T-cells in PH seems to be as polyvalent as the functional repertoire of T-cells in general. While stereotypical functions like the anti-inflammatory capacities of T_regs_ and the pro-autoimmune properties of T_H_17 cells also seem to hold true in PH, there still is a prominent lack of knowledge with regards to a deeper understanding of molecular mechanisms triggering an altered T-cell appearance, the interplay of different T-cell subsets and the communication of T-cells with other compartment of the immune system in PH. Nonetheless, first potential specific targets like IL-17, T_regs_ or ROCK highlight the value of such investigations, which could ultimately advance the treatment of dysregulated immunity as driver of PH.

## Conclusion

Multiple components and cells of the innate and adaptive immunity bear essential contribution to the pathophysiological scaffolding of PH in terms of vascular remodeling and aggravation of the PH phenotype. Pathological dysregulation, in particular elevation of, e.g., monocytes, macrophages, DC subsets and T_H_17 cells and decreased NK cells, T_regs_ as well as formation of autoantibodies have been observed in multiple models of PH but foremost animal models, stressing the need for further human studies including different types of PH. At the same time, a deeper insight into the molecular mechanisms and interactions of the depicted contributors to the pathophysiology of PH is of essential need to lead the way for specific targeted therapeutic approaches intervening into the affected homeostasis of innate and adaptive immunity. As highlighted in this review, a vast amount of potential and promising targets are on hand. Considering therapeutic options in other diseases with underlying immune dysregulation and autoimmune character, specific immunomodulators have already been introduced, which give hope to be put to use also as effective treatments of PH. Exemplarily, neutralization of TNF-*α* like in patients with rheumatoid arthritis by use of TNF-α type 2 receptor-IgG1 fusion protein (etanercept) or chimeric monoclonal antibody against TNF-α (infliximab; Lipsky et al., 2000) might oppose TNF-α signaling and hence inflammation and subsequent vascular remodeling in PH. Besides, the attempts of evoking B-cell depletion through a mononuclear anti-CD20 antibody (rituximab; Zamanian et al., 2021) seem worth to pursue considering the successful treatment of autoimmune diseases like ANCA-associated vasculitis with rituximab (Hassan and Gaffo, 2017). Further, biologics targeting the IL-17 receptor (brodalumab) or neutralizing IL-17 (ixekizumab and secukinumab) are highly effective in the treatment of psoriasis (Balato et al., 2017) and could be implemented in preclinical trials of PH. With respect to the complexity of detecting the right time point of treatment and the group of patients who might benefit from immunomodulation, it will, nevertheless, remain a challenge to establish reliable clinical treatment regimes. However, the success of targeted anti-inflammatory therapeutic strategies in a wide range of immune modulated diseases encourages to draw on effective applicable therapeutic approaches including personalized medicine in the future.

## Author Contributions

TF-H, FB, JG, and SS have substantially contributed to the conception or design of the work, designed the figures, drafted the article, and revised it critically for important intellectual content. All authors agreed to be accountable for all aspects of the work in ensuring that questions related to the accuracy or integrity of any part of the work are appropriately investigated and resolved. All authors contributed to the article and approved the submitted version.

## Funding

TF-H was supported by the Berlin Institute of Health (BIH). FB was supported by the BIH-MD-TRENAL medical research student stipend. JG was supported by the German Centre for Cardiovascular Research (DZHK). SS was supported by the German Foundation of Heart Research.

## Conflict of Interest

The authors declare that the research was conducted in the absence of any commercial or financial relationships that could be construed as a potential conflict of interest.

## Publisher’s Note

All claims expressed in this article are solely those of the authors and do not necessarily represent those of their affiliated organizations, or those of the publisher, the editors and the reviewers. Any product that may be evaluated in this article, or claim that may be made by its manufacturer, is not guaranteed or endorsed by the publisher.
